# Pleuroparenchymal Fibroelastosis as a Late-Onset Pulmonary Toxicity after Treatment with Anticancer Chemotherapy for High-Risk Neuroblastoma

**DOI:** 10.1155/2024/4352032

**Published:** 2024-05-18

**Authors:** Satoshi Yokoyama, Risa Kanai, Daisuke Fukao, Keigo Hamahata

**Affiliations:** ^1^Department of Pediatric Surgery, Japanese Red Cross Society, Wakayama Medical Center, 4-20 Komatsubara-dori, Wakayama 640-8558, Japan; ^2^Department of Pediatrics, Japanese Red Cross Society, Wakayama Medical Center, 4-20 Komatsubara-dori, Wakayama 640-8558, Japan

## Abstract

Pleuroparenchymal fibroelastosis (PPFE) is a rare, progressive, restrictive lung disease characterized by hypercarbic respiratory failure. In pediatrics, it has been described in patients with a history of malignancy who have received a bone marrow transplant, chemotherapy, or radiotherapy. It is characterized by pleural thickening, fibrosis, subpleural elastosis, and intraalveolar collagen deposits. Survival is poor, and the only therapy is lung transplantation. Here, we report a patient who developed PPFE as a late-onset pulmonary toxicity after treatment with anticancer chemotherapy for high-risk neuroblastoma (NB).

## 1. Introduction

Pleuroparenchymal fibroelastosis (PPFE), a rare, progressive, restrictive lung disease, is characterized by hypercarbic respiratory failure. In pediatrics, the condition is characterized by pleural thickening, fibrosis, subpleural elastosis, and intraalveolar collagen deposits, and cases have been reported in patients with malignancy who have received a bone marrow transplant, chemotherapy, or radiotherapy. Lung transplantation remains the only therapy, however, and survival is poor [1–5]. Here, we report a patient who developed PPFE with pectus excavatum, which is considered a late-onset pulmonary toxicity after multiple chemotherapies for high-risk neuroblastoma (NB).

## 2. Case Presentation

The patient was diagnosed with stage four, high-risk retroperitoneal NB at age 1.5 years. Treatment consisted of high-risk NB treatment per the Japan Childhood Cancer Group Neuroblastoma Committee (JNBSG) protocol JN-H-07. She underwent five cycles of induction chemotherapy including cyclophosphamide, vincristine, pirarubicin, and cisplatin followed by high-dose chemotherapy with hematopoietic stem cell rescue (HDC) using peripheral blood stem cells as consolidation therapy which also included melphalan, etoposide, and carboplatin. The primary tumor was surgically removed after induction therapy and HDC. The postoperative radiotherapy for the primary tumor site was performed.

**Figure 1:**
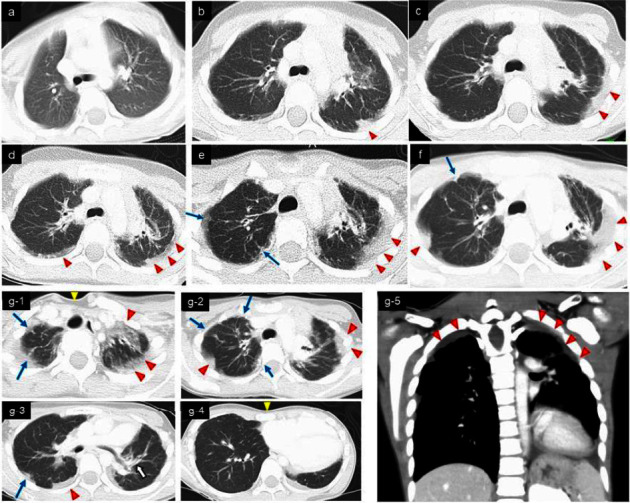
Chest CT images show progressive pleural and pulmonary fibrosis/scarring consistent with PPFE: ((a) 2015 (at the time of discharge), (b) 2017, (c) 2018, (d) 2019, (e) 2020, (f) 2021, and (g) 2022 (at the age of 9 years)). CT image shows pleural thickening (red arrow heads) and bronchiectasis (white arrow), a reticular shadow (blue arrows) localized in the bilateral upper lobes predominant. Pectus excavatum (yellow arrows) is also observed.

**Figure 2:**
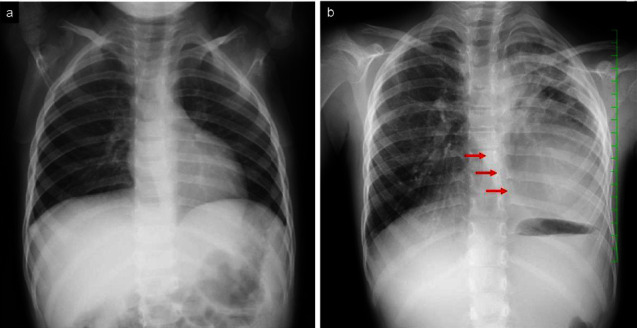
(a) Chest radiography at 2 years of age (at the time of discharge). (b) Mediastinal shift to the left side due to the volume loss of left lung (red arrows) at the age of 9 years.

**Figure 3:**
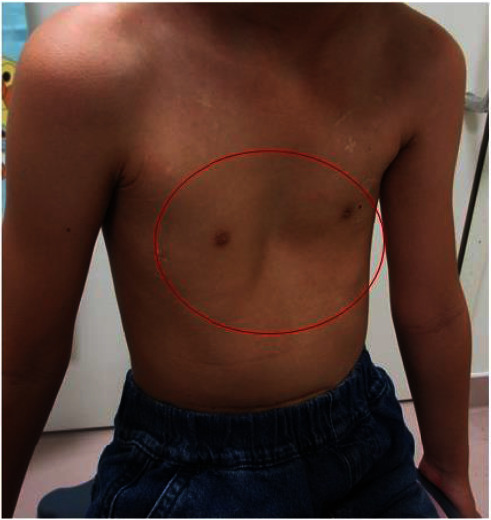
She demonstrates pectus excavatum at the age of 9 years.

**Figure 4:**
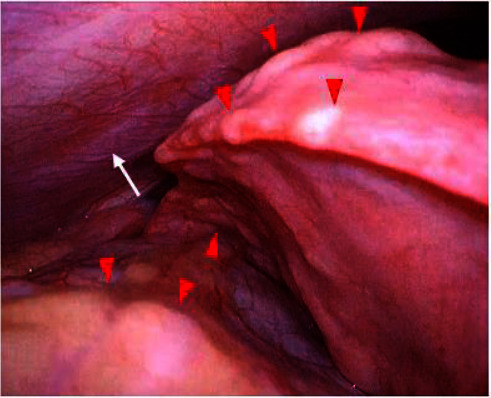
Thoracoscopy shows left upper lobe-predominant subpleural and perilobular fibrosis (red arrow heads). There was no evidence of pleural thickening or inflammatory changes in the parietal pleura (white arrow).

She has since remained in first complete remission. However, she also developed sensorineural hearing loss and growth retardation associated with multidrug chemotherapy for high-risk NB. Two years after onset, follow-up chest computed tomography (CT) showed thickening of the pleura in the bilateral upper lobes although she did not show any symptoms (Figures 1(a) and 1(b)). She complained of gradually worsening dry cough, shortness of breath, and dyspnea on exertion. During the next 5 years, regular chest roentgenogram revealed increased pulmonary infiltrates, volume loss predominantly in the upper lung, pleural thickening, and severe mediastinal shift (Figures 2(a) and 2(b)). CT demonstrated pectus excavatum, pleural thickening, and a reticular shadow in the bilateral upper lobes (Figures 1(a)–1(g)). Physical examination demonstrated pectus excavatum which was not evident at a diagnosis of NB (Figure 3). The patient's body mass index (BMI) was 12.80 (kg/m^2^). Laboratory blood tests and blood gas analysis were within normal range. Krebs von den Lungen-6 (KL-6) was 514 U/mL (normal <500 U/mL). Pulmonary function test (PFT) showed severe restrictive ventilatory impairment, with a forced vital cavity (FVC) of 0.47 L (32.9% predicted), forced expiratory volume in one second (FEV1) of 0.46 L (35.4% predicted), and FEV1/FVC ratio (FEV1%) of 97.8%. Given the unclear diagnostic picture, the patient underwent left-sided video-assisted exploratory thoracoscopy. The left upper lobe predominant indicated fibrous-appearing thickening of the peripheral aspect of the parenchyma. There was no evidence of pleural thickening or inflammatory changes in the parietal pleura at thoracoscopy (Figure 4). Lung biopsy was not performed due to the risk of refractory postbiopsy pneumothorax. A diagnosis of PPFE was made, possibly as a late complication after chemotherapy for NB.

After diagnosis, the patient was started on nutritional therapy, respiratory rehabilitation, and antifibrotic medication (pirfenidone) and currently remains clinically and functionally stable. She is under a careful follow-up with a view to future lung transplantation.

## 3. Discussion

In this case study, we report a patient with multiple chemotherapy for initially treated for high-risk NB who developed PPFE with pectus excavatum.

Survival rates for most childhood malignancies have significantly improved over the last few decades. Given the long-life expectancy of childhood cancer patients, however, attention has now focused on the late sequelae of cancer treatments on vital organ function, particularly cardiac and pulmonary conditions and secondary cancer [6].

PPFE is among a newly classified group of entities termed idiopathic interstitial pneumonias. Unlike idiopathic PPFE, which is relatively common in adults, secondary PPFE is considerably more common in children, in whom it is typically associated with a distant history of cancer therapy. PPFE is characterized by pleural and subpleural parenchymal thickening due to elastic fiber proliferation with minimal inflammation and most commonly presents with dyspnea, cough, and/or pneumothorax, although weight loss, hypoxemia, respiratory failure, and hypercarbia may also be present. The clinical course of PPFE is progressive and prognosis is poor [2, 3]; indeed, an effective treatment is not available, with most patients with advanced disease requiring lung transplantation [4, 5].

Although the etiology of secondary PPFE is unclear, possibilities include chemotherapeutic agents, respiratory infection, cell-mediated immune responses, either alone or in combination [7–9]. Nguyen reported a mean time between chemotherapy and radiation therapy [2] and/or bone marrow transplantation and clinical presentation of PPFE of 8.4 years (range: 5.6–12.1 years), consistent with the 7 years in our present case. In their series of PPFE, NB was the most commonly associated childhood malignancy [9], raising the possibility that some cases of restrictive lung disease reported in NB survivors are due to undiagnosed PPFE.

With regards to diagnosis of PPFE in pediatric patients, comprehensive evaluation should include a medical history, physical examination, lab tests, pulmonary function tests, and imaging. Lung biopsy may be required. When the clinical history supports a diagnosis of PPFE, pulmonary function testing, radiography, and biopsy-mediated histopathological confirmation are unnecessary and may in fact be detrimental. In particular, the risk of extensive subpleural fibrosis and elastin deposition is reportedly increased in patients with PPFE, which in turn places them at increased of iatrogenic pneumothorax, a well-known complication of lung biopsy [7, 10].

Regarding management, evidence-based guidelines for PPFE specific to pediatric patients are limited. While antifibrotic agents such as pirfenidone and nintedanib play a key role in PPFE management in older adults owing to their demonstrated efficacy in reducing disease progression and preserving lung function, their effectiveness and safety in children awaits further evaluation, particularly with regard to age-related differences in long-term effects and safety [11, 12]. Supportive measures are essential regardless of the patient age. Travis et al. report the importance of pulmonary rehabilitation programs, including exercise training, education, and psychosocial support, and these can improve exercise tolerance, dyspnea, and quality of life. Treatment options such as antifibrotic therapy, supportive measures, and lung transplantation should be selected based on individual clinical characteristics, disease severity, and potential risks and benefits. Optimization of management and outcomes requires close monitoring of disease progression, lung function, and quality of life [13].

Marked progress over the past two decades in understanding the biology of NB has led to a refining of risk stratification and treatment modifications, as reflected in increases in 5-year survival in affected children. Nevertheless, survivors are at risk for various treatment-related complications, or “late effects” [14], which in the respiratory system include pulmonary chronic respiratory symptoms, abnormal PFT, and bronchiectasis. PPFE may be presumed with clinical presentation of restrictive lung disease, dyspnea, cough, or spontaneous pneumothorax years after treatment for NB, combined with platythorax, upper lobe pleural and septal thickening, and bronchiectasis on chest CT. The improving survival from NB has raised awareness of the late pulmonary complications of NB therapy [15].

This case highlights the need for particular attention to PPFE as a late complication after treatment for NB. Early detection of late complications and therapeutic intervention are important in the long-term follow-up of NB, but subjective symptoms are nonspecific, and early recognition is difficult and requires attention.

## Data Availability

The clinical data used to support the findings of this study are included within the article.

## Abbreviations

PPFE: Pleuroparenchymal fibroelastosis
NB: Neuroblastoma.
